# Epigallocatechin-3-gallate mitigates cerebral ischemia-reperfusion injury by promoting microglia toward M2 phenotype via Nrf2/HO-1 pathway

**DOI:** 10.1038/s41598-026-60227-0

**Published:** 2026-06-28

**Authors:** Yan Zhang, Yalin Huang, Guanfeng Xie, Chao Qin, Shizhu Chen

**Affiliations:** 1 Department of Rehabilitation Medicine, Jiangbin Hospital of Guangxi Zhuang Autonomous Region, Nanning, Guangxi China; 2 Department of Rehabilitation Medicine, The Second Affiliated Hospital of Guangxi Medical University, Nanning, Guangxi China; 3 Department of Neurology, The First Affiliated Hospital of Guangxi Medical University, Nanning, Guangxi China

**Keywords:** Ischemic stroke, Epigallocatechin-3-gallate, Cerebral ischemic-reperfusion injury, Microglia, Ferroptosis, Diseases, Neurology, Neuroscience

## Abstract

Ischemic stroke remains the leading disease in terms of global disability and fatality rates. Cerebral ischemia-reperfusion injury (CIRI) and neuroinflammation induced by CIRI represents a challenge for clinical therapy of ischemic stroke. Epigallocatechin-3-gallate (EGCG), the predominant catechin in green tea, is known to mitigate ischemic stroke and inflammation, while its role in regulating neuroinflammation post-recanalization remains unclear. This study aimed to explore the effect and mechanism of EGCG on the regulation of CIRI. The CIRI in vivo model was established by middle cerebral artery occlusion/reperfusion (MCAO/R) in rats and EGCG was administered via intracerebroventricular injection before MCAO/R. The neurological scores, infarct volume, neuronal injury, and microglial biomarkers were evaluated. BV2 cells subjected to oxygen–glucose deprivation/reoxygenation (OGD/R) were used to establish an in vitro CIRI model. After pretreatment with EGCG and/or ML385, OGD/R, cell viability, microglial biomarkers, pro-inflammatory and anti-inflammatory factors, and transcription factor nuclear factor erythroid 2-related factor 2 (Nrf2) and hemeoxygenase-1 (HO-1) were detected. Different conditioned media collected from BV2 cells were used for culturing the neuronal mimic cell line SH-SY5Y. EGCG administration in vivo significantly improved neurological deficits, infarct volume, and neuronal damage. Moreover, pretreatment with EGCG significantly increased BV2 cells viability and anti-inflammatory factors, and reduced toxicity and ferroptosis in SH-SY5Y cells. Furthermore, the upregulation of M2 markers was consistent with in vivo experiments. However, this protective effect was abolished by ML385, an Nrf2 inhibitor. EGCG upregulated Nrf2 and HO-1 protein expression, thus promoting microglial polarization toward the M2 phenotype. EGCG has a protective effect on CIRI by promoting the polarization of microglia toward the M2 phenotype via the Nrf2/HO-1 pathway to inhibit ferroptosis of neuron, which may provide a novel target for ischemic stroke treatment.

**Supplementary Information:**

The online version contains supplementary material available at 10.1038/s41598-026-60227-0.

## Introduction

Ischemic stroke is a leading cause of disability and death worldwide. Vascular recanalization is the most important method for rescuing the ischemic penumbra and improving prognosis^1^. However, reperfusion injury followed by vascular recanalization, defined as cerebral ischemia-reperfusion injury (CIRI), presents a challenge for clinical therapy of ischemic stroke^2^. The peroxidation environment formed by reperfusion leads to overactivation of microglia and induces an inflammatory cascade reaction that leads to adenosine triphosphate (ATP) deficiency, dysfunction of ion-exchange channels, and retention of reactive oxygen species (ROS)^2^. However, despite several studies on CIRI, an effective treatment is unavailable. Therefore, agents targeting CIRI may serve as novel therapeutic interventions for ischemic stroke. Previous studies have suggested that spontaneous intracranial sterile inflammation after ischemic stroke is an important factor in CIRI that leads to poor prognosis^3^. Microglia, the resident immune cells in the central nervous system (CNS), have been the focus of many studies. However, eradication of microglia to alleviate the inflammatory response following stroke has remained unsuccessful, and instead, the removal of microglia has expanded the injury range of ischemic stroke^4,5^. Activated microglia are classified into the M1 and M2 phenotypes. The M1 phenotype releases multiple pro-inflammatory cytokines and exacerbates brain damage; whereas M2 phenotype significantly promotes brain repair and neuronal regeneration^6^. Therefore, drugs that promote microglial polarization toward M2 play a vital role in the treatment of ischemic stroke.

Epigallocatechin-3-gallate (EGCG), the most abundant polyphenol in tea, is one of the most widely studied tea components^7^. EGCG has been reported to have therapeutic potential against various human diseases, including cancer, cardiovascular diseases, and coronavirus disease^8–10^. Moreover, recent studies suggest that EGCG has anti-inflammatory effects in the CNS^11^, indicating that EGCG may regulate spontaneous inflammation after stroke. Nevertheless, previous studies on EGCG in ischemic stroke have primarily focused on its antioxidant effects during the ischemic phase, while its therapeutic potential during CIRI remains poorly characterized^12,13^. Elucidating this mechanism could expand the clinical applications of EGCG and provide more robust evidence for its therapeutic indications.

The nuclear factor-erythroid 2 (NF-E2) p45-related factor 2 (Nrf2)/hemeoxygenase-1 (HO-1) signaling pathway mainly inhibits progression of inflammation by regulating anti-inflammatory gene expression^14^. Remarkably, Nrf2/HO-1 pathway belong to be a downstream target of EGCG^15^. Moreover, the Nrf2/HO-1 pathway is strongly linked to macrophage/microglial phenotypic polarization^16–18^. Therefore, EGCG-mediated regulation of the Nrf2/HO-1 pathway may play a crucial role in CIRI. Further investigation is necessary.

Therefore, in the present study, we aimed to explore the effect of EGCG on microglial polarization in the CIRI models and uncover the underlying mechanism on neuroprotection.

## Materials and methods

### Animals and ethics statement

Adult male Sprague-Dawley (SD) rats weighing 250–280 g were obtained from the Experimental Animal Center of the Guangxi Medical University. The rats were maintained in a sterile environment at 25 ± 2 ℃ under 12 h light/dark cycle. All animal experiments were approved by the Animal Care and Welfare Committee of the Guangxi Medical University (Project proposal number: 202109003) and were performed in accordance with the ARRIVE guidelines.

### Animal model of transient focal cerebral ischemia and experimental design

Rats were deprived food, but were provided free access to water overnight before surgery. Rats were anesthetized by intraperitoneal injection of pentobarbital (40 mg/kg) and placed on the operating table. As previously described^19^, the CIRI model was established using middle cerebral artery occlusion/reperfusion (MCAO/R) operation. After exposing the right common carotid artery (CCA), internal carotid artery (ICA), and external carotid artery (ECA), the CCA was temporarily blocked using a vascular clip. After ligating the ECA, a 4/0 monofilament nylon suture (RWD Life Science Co., Ltd.) was inserted from the ECA to ICA to block blood flow in the middle cerebral artery. After 2 h, the monofilament nylon sutures were retracted. The rectal temperature of the rats remained at 37.0 ± 0.5 °C during surgery. Furthermore, reperfusion was allowed for 24 h. The sham-operation group did not receive a monofilament, but underwent the same surgery.

Drug treatment was performed immediately before MCAO surgery. A 5 µL of 0.5 mg/mL or 1.0 mg/mL EGCG (A2600, APExBIO, USA) was used for intracerebroventricular injection in rats. Briefly^20^, anesthetized rats were fixed according to stereotaxic instructions (RWD Life Science Co., Ltd.). A scalp incision was made, and a hole was drilled 1.8 mm lateral and 1.0 mm posterior to the right bregma. The drug was then injected using a microinjection pump at 4.2 mm depth below the endocranium. An intraperitoneal injection of edaravone (SE8870, Solabiar, Beijing, China) (3 mg/kg)^21^ was used to assess the protective effect of EGCG. A 1% DMSO (5 µL) was injected into the right lateral ventricle in the vehicle group. One hundred male SD rats were randomly divided into the following five groups (*n* = 20 each)—Sham+vehicle group; MCAO/R+vehicle; MCAO.R+EGCG (0.5 mg/mL); MCAO/R+EGCG (1.0 mg/mL); and MCAO/R+edaravone. After treatment, rats were administered an overdose of phenobarbital sodium (200 mg/kg) via intraperitoneal injection.

### Cell culture

The mouse microglial cell line BV2 and human neuroblastoma cell line SH-SY5Y were obtained from the Chinese Academy of Sciences. High-glucose Dulbecco’s Modified Eagle’s Medium (DMEM) (C11995500BT, Gibco, USA) and DMEM F12 (10565018, Gibco, USA) were used for culturing BV2 and SH-SY5Y cells, respectively. Additionally, media were supplemented with 10% fetal bovine serum (GB-106, Gibco, USA) and 1% penicillin–streptomycin. Cells from generations 5–20 were used for follow-up experiments when the density reached 70%.

### Oxygen–glucose deprivation and reoxygenation (OGD/R) and cell grouping

Briefly, high-glucose DMEM was removed and replaced with glucose-free DMEM (11966025, Gibco, USA). BV2 cells were then placed in a modular incubator filled with 95% N_2_/5% CO_2_ for 6 h at 37 ℃. Further, the glucose-free DMEM was replaced with high-glucose DMEM, and the BV2 cells were cultured for another 24 h in a normal incubator with 5% CO_2_ at 37 ℃^19^.

We selected 0.5, 2.0, 5.0, 10.0, and 15.0 µmol/L concentrations of EGCG to pretreat BV2 cells for 24 h followed by OGD/R. Furthermore, cells were incubated with the Nrf2-specific inhibitor ML385 (B8300, APExBIO, USA) (1 µmol/L)^22^ for 30 min before EGCG intervention in the ML385 + EGCG + OGD/R group. A 5 µL of 1% DMSO was used in the vehicle group.

To completely evaluate the effect of EGCG on CIRI, we used BV2 cells’ supernatant with different treatments as conditioned media for the SH-SY5Y cells. The supernatant of BV2 cells treated with DMSO, EGCG, and/or ML385 following OGD/R was collected. The SH-SY5Y cells were plated in 96-well plates at a density of 10^3^ cells/well, the medium was replaced with microglial-conditioned medium, and cultured for another 24 h^23^.

### Neurological function

The modified Longa score was determined by a blinded investigator^24^. Briefly, no deficits were defined as “0”; the contralateral forelimb could not be fully extended was defined as “1”; no extension of the contralateral forelimb was defined as “2”; mild circling was defined as “3”; severe circling was defined as “4”; and falling to the contralateral side was defined as “5.”

The corner test was performed according to established protocols to evaluate asymmetries in turning behavior^24^. Left-turn frequency was recorded during daily sessions consisting of 10 consecutive trials for quantitative assessment of behavioral asymmetries.

### Measurement of the infarct area

The cerebral infarct volume was measured after 24 h of reperfusion. Briefly, rats were anesthetized with 4% pentobarbital, and the brains were rapidly dissected and cut into coronal segments (2 mm thickness). Further, the segments were incubated with 2% 2, 3, 5-triphenyltetrazolium chloride (TTC) (Sigma-Aldrich, St. Louis, MO) for 20 min at 37 ℃. ImageJ software was used to analyze the images of TTC-stained segments. The cerebral infarct volume data are presented as: ([contralateral volume]-[ipsilateral undamaged volume])/contralateral volume × 100^19^.

### Nissl staining

The brains of rats in each group were fixed in 4% paraformaldehyde for 24 h, dehydrated, and embedded in paraffin. Subsequently, the brain was cut into 5 μm serial sections. After dewaxing and rehydration, slices were stained with Nissl solution (G1036, Serviobio, Wuhan) for 2–5 min. All operations followed the manufacturer’s instructions. One view of the hippocampal CA1 area and three views of the cortex in the same section were randomly selected. Image-Pro Plus 6.0 was used for analyzing the surviving neurons^24^.

### Immunofluorescence staining

The tissue sections or cells were fixed with 4% paraformaldehyde for 30 min and incubated with 0.25% Triton X-100 for 20 min at 25 °C. Further, sections or cells were blocked with 10% goat serum, followed by incubation with the following primary antibodies: rabbit polyclonal anti-CD206 (1:200, PA5-101657, Intrivogen, Carlsbad, CA), rabbit polyclonal anti-CD32 (1:200, PA5-95557, Intrivogen, Carlsbad, CA), rabbit polyclonal anti-iNOS (1:250, PA1-038, Intrivogen, Carlsbad, CA), rabbit polyclonal anti-Arginase-1 (1:250, PA5-85267, Intrivogen, Carlsbad, CA), rabbit monoclonal anti-phospho-NF-κB p65 (1:100, #3033, CST, USA), and mouse monoclonal anti-Iba1 (1:250, GB12105, Servicebio, Wuhan, China) at 4 ℃. After 12 h incubation, goat anti-rabbit (1:500, SA5-35571, Invitrogen, Carlsbad, CA) and goat anti-mouse (1:500, SA5-35521, Invitrogen, Carlsbad, CA) secondary antibodies were applied. Finally, DAPI (S2110, Solarbio, Beijing, China) was added as a nuclear marker. The sections were visualized under a fluorescence microscope (OLYMPUS, Japan), and data were analyzed using ImageJ software.

### Western blotting

The brain tissue sections and BV2 cells were lysed with RIPA buffer. The samples were centrifuged, and the supernatant was collected. After quantification by the BCA method, proteins were denatured by boiling for 10 min, separated using 8–12% SDS-PAGE, and transferred to PVDF membranes. The membranes were blocked with 5% defatted milk, and incubated with the following primary antibodies: rabbit polyclonal anti-iNOS (1:2,500, PA1-038, Invitrogen, Carlsbad, CA), rabbit polyclonal anti-Arginase-1 (1:2,500, PA5-85267, Invitrogen, Carlsbad, CA), rabbit polyclonal anti-Nrf2 (1:2,000, AF7006, Affinity, US), rabbit polyclonal anti-HO-1 (1:2,500, ab13248, Abcam, UK), rabbit polyclonal anti-GPX4 (1:2,500, DF6701, Abcam, UK), rabbit polyclonal anti- xCT/SLC7A11 (1:2000, 98051 S, Abcam, UK), rabbit polyclonal anti-ACSL4 (1:2,500, TD12141S, Abmart, China), rabbit polyclonal anti-COX2 (1:2000, ab283574, Abcam, UK), rabbit polyclonal anti-tubulin (1:3,000, 26195-1-AP, Proteintech, Wuhan, China), rabbit polyclonal anti-β-actin (1:2,500, GB11001, Servicebio, Wuhan, China), and polyclonal anti-LaminB1 (1:2,500, 12987-1-AP, Proteintech, Wuhan, China). After three washes with 1× TBST, the membranes were incubated with HRP-conjugated goat anti-rabbit secondary antibody (1:10,000, SA00001-2, Proteintech, Wuhan, China). Enhanced chemiluminescence (ECL) reagent was used to visualize the protein bands that were quantified using ImageJ software.

### Cell viability

The viability of BV2 and SH-SY5Y cells was assessed using Cell Counting Kit-8 (CCK-8) assay (CK04-500, DOJINDO, JAPAN). All steps were performed according to the manufacturer’s instructions. The blank group referred to an equal volume of medium without cells. After treatment, as described above, 10 µL of CCK-8 solution was added to each well and incubated for 2 h in dark at 37 ℃. Absorbance was measured using a microplate reader (Thermo Scientific, USA) at 450 nm wavelength. We used the following formula to calculate cell viability: Cell viability (%) = (experimental group-blank group)/(control group-blank group)×100.

### Enzyme-linked immunosorbent assay (ELISA)

The release of pro-inflammatory and anti-inflammatory factors in BV2 cells was detected using the double-antibody sandwich method. All steps were performed according to the manufacturer’s instructions. Cell medium of each group was collected and centrifuged at 1,000 ×*g* at 4 ℃ for 20 min, and 100 µL of supernatant was collected. A mouse tumor necrosis factor-α (TNF-α) ELISA Kit (E-MESL-M0002, Elabscience), interleukin (IL)−1β ELISA Kit (E-MESL-M0003, Elabscience), IL-4 ELISA Kit (E-EL-M0043c, Elabscience), and IL-10 ELISA Kit (E-EL-M0046c, Elabscience) were used for detection. The concentrations of these factors were calculated based on a standard curve and were expressed in pg/mL.

### Reactive oxygen species (ROS) detection

ROS levels were detected using ROS Assay Kit (TH615, DOJINDO, JAPAN). All procedures were performed according to the manufacturer’s instructions. Briefly, the cells were inoculated into 96-well plates. When the density reached 70%, the cells were washed twice with Hank’s Balanced Salt Solution. Further, the working solution was added. After incubation in an incubator filled with 5% CO_2_ at 37℃ for 30 min, the cells were washed three times with Hank’s Balanced Salt Solution. The results were recorded using a fluorescence microscope (OLYMPUS, Japan).

### Statistical analysis

Statistical analyses were performed using SPSS software (version 19.0). All experiments were repeated at least three independent times. Data are presented as mean ± SEM, and statistical analysis is performed using one-way ANOVA test followed by Tukey’s multiple comparisons test when P value of Levene test > 0.05 or Welch test followed by Dunnett T3 multiple comparisons test when the P value of Levene test < 0.05. Normality and variance are assessed via Shapiro-Wilk test and Levene’s test, respectively.

## Results

### EGCG alleviated the neurological deficits and reduced the brain injury in MCAO/R rats

**Fig. 1:**
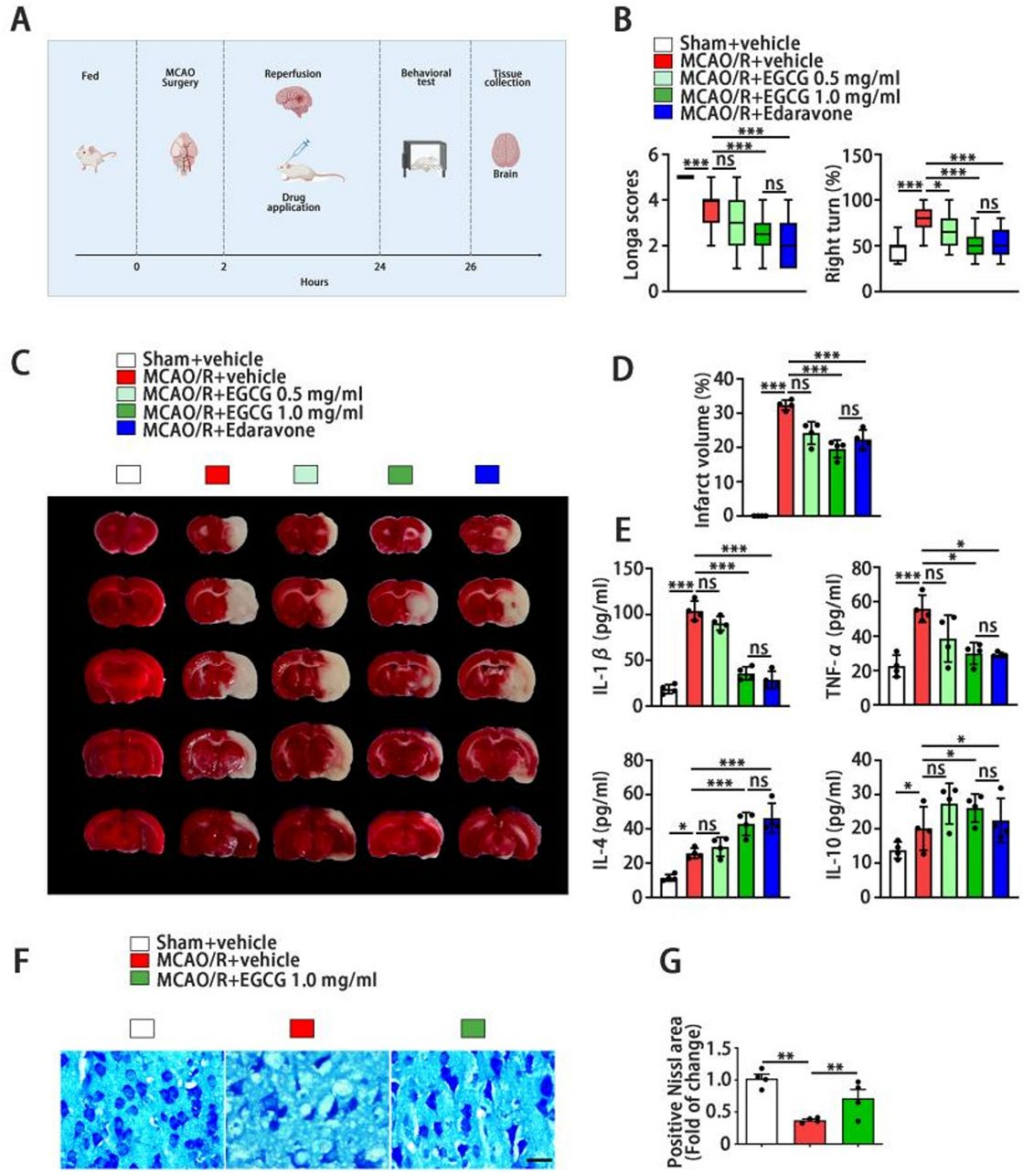
EGCG Ameliorates Cerebral Ischemic-Reperfusion Injury in MCAO/R rats. **(A)** Schematic diagram. **(B)** Modified Longa score test and Corner test, *n* = 20. **(C, D)** Representative images of TTC staining and quantification of infarct volume, *n* = 4. **(E)** Brain proinflammatory and anti-inflammatory detected by ELISA, *n* = 4. **(F**, **G)** Representative images and quantification of Nissl staining, *n* = 4. Scar bar = 25 μm. Data are presented as mean ± SEM, and statistical analysis is performed using one-way ANOVA test followed by Tukey’s multiple comparisons test when P value of Levene test > 0.05 or Welch test followed by Dunnett T3 multiple comparisons test when the P value of Levene test < 0.05. Normality and variance are assessed via Shapiro-Wilk test and Levene’s test, respectively. ^*^ P *< 0.05*, ^**^ *P* < 0.01, ^***^ *P* < 0.001.

The relationship between EGCG treatment and duration of post-ischemia-reperfusion in rats was evaluated (Fig. 1A). As indicated by Longa scores and corner test results, the administration of 1.0 mg/mL EGCG significantly improved neurological function in MCAO/R rats, exhibiting neuroprotective effects equivalent to the classical antioxidant drug edaravone (an effective oxygen free radical scavenger, can alleviate CIRI) (Fig. 1B). Furthermore, TTC staining revealed that 1.0 mg/mL EGCG significantly reduced infarct volume, with efficacy similar to edaravone treatment (Fig. 1C-D). Interestingly, the ELISA showed that both 1.0 mg/mL EGCG and edaravone significantly reduced pro-inflammatory cytokines (IL-1β and TNF-α) while upregulating anti-inflammatory factors (IL-4 and IL-10) in the brains of MCAO/R rats (Fig. 1E). Additionally, the Nissl staining showed that 1.0 mg/mL EGCG significantly ameliorated the brain injury of rats subjected to MCAO/R (Fig. 1F-G). These results revealed that 1.0 mg/mL EGCG significantly ameliorated MCAO/R injury in rats by regulating neuroinflammation.

### EGCG promoted microglia polarization toward M2 phenotype in CIRI

**Fig. 2:**
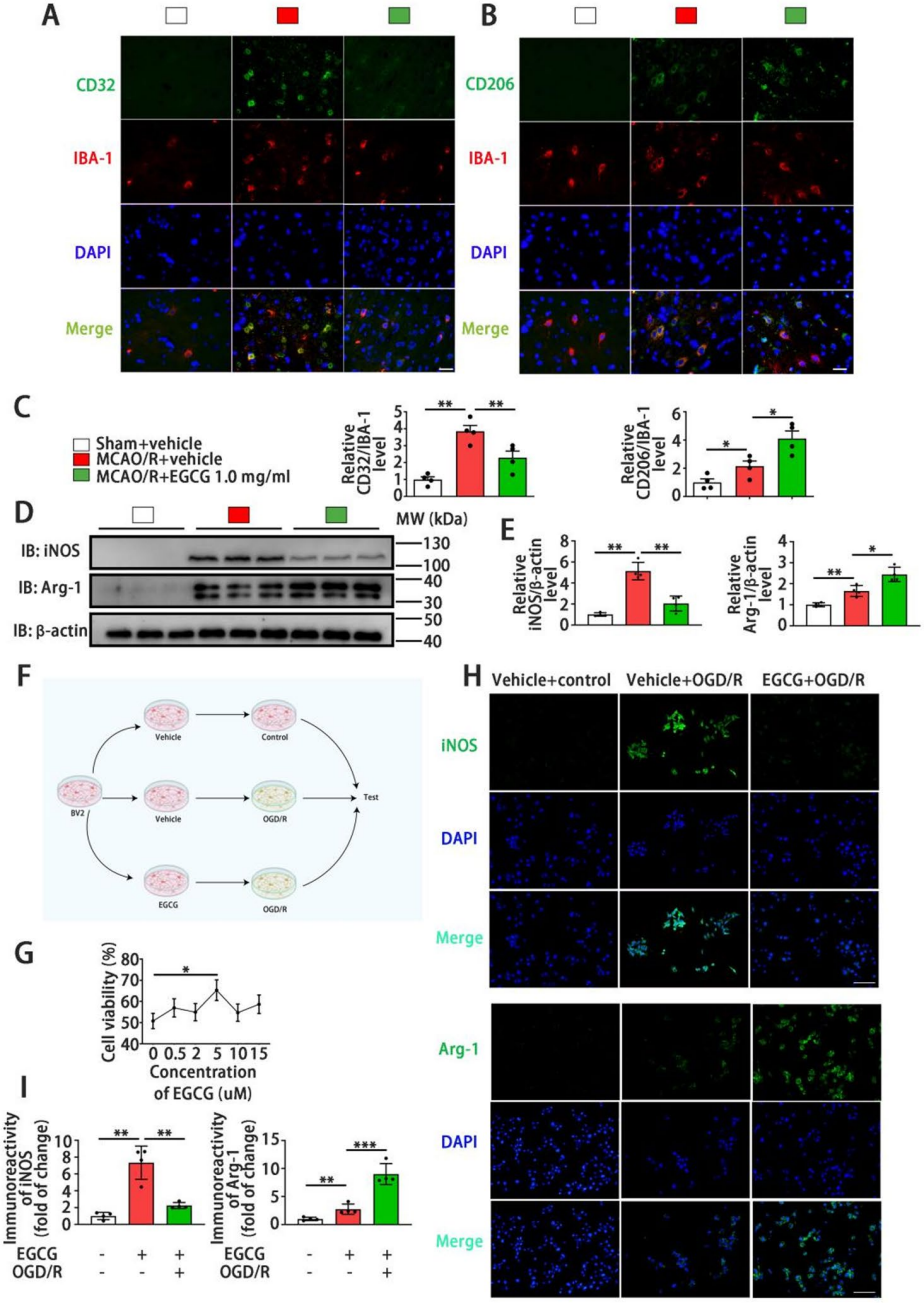
EGCG Promotes Microglial Polarization Toward the M2 Phenotype following CIRI. **(A–C)** Immunofluorescence staining for CD32 or CD206 (green) and for IBA-1 (red) in the infarct area, *n* = 4. Scar bar = 20 μm. **(D**, **E)** Western blot analysis of iNOS and Arg-1, *n* = 4. **(F)** Schematic diagram of in vitro model. **(G)** Cell viability detected by the CCK8 assay, *n* = 10. **(H**–**I)** Immunofluorescence staining for iNOS and Arg-1 of BV2 cells, *n* = 4. Scar bar = 100 μm. Data are presented as mean ± SEM, and statistical analysis is performed using one-way ANOVA test followed by Tukey’s multiple comparisons test when P value of Levene test > 0.05 or Welch test followed by Dunnett T3 multiple comparisons test when the P value of Levene test < 0.05. Normality and variance are assessed via Shapiro-Wilk test and Levene’s test, respectively. ^*^ P *< 0.05*, ^**^ *P* < 0.01, ^***^ *P* < 0.001.

Based on the findings above, we sought to identify the effects about how EGCG regulate neuroinflammation in MCAO/R rats. CD32 and CD206 are established markers of M1 and M2 microglial subtypes, respectively^25,26^. Immunofluorescence analysis revealed significantly higher levels of CD32-positive microglial cells in the infarct area of the MCAO/R+vehicle group compared to the Sham+vehicle group. In contrast, CD32 expression was markedly reduced in the MCAO/R+EGCG group relative to the MCAO/R+vehicle group (Fig. 2A and C). Additionally, EGCG treatment significantly increased CD206 levels (Fig. 2B-C). We next examined phenotype-associated functional proteins. Inducible nitric oxide synthase (iNOS), expressed in the brain following ischemic injury, mediates TNF-α production and NF-κB pathway activation in M1 microglia and contributes to mitochondrial dysfunction and glutamate excitotoxicity. While MCAO/R significantly increased iNOS expression, EGCG pretreatment effectively suppressed this response (Fig. 2D-E and Supplementary file 1). Arginase-1 (Arg-1), an M2 microglia/macrophage marker, promotes neuroprotection through tissue remodeling and behavioral recovery after stroke. Western blot analysis showed significantly higher Arg-1 expression in the MCAO/R+EGCG group compared to the MCAO/R+vehicle group (Fig. 2D-E and Supplementary file 1).

To further validate EGCG’s protective effects, we employed an in vitro OGD/R model using BV2 cells (Fig. 2F). Remarkably, EGCG attenuated OGD/R-induced cytotoxicity and increased BV2 cell viability (Fig. 2G). Consistent with the in vivo findings, EGCG suppressed iNOS while upregulating Arg-1 expression in OGD/R-treated BV2 cells (Fig. 2H-I). These results collectively demonstrate that EGCG drives microglial polarization toward the anti-inflammatory M2 phenotype during CIRI.

### EGCG promotes microglial M2 polarization via Nrf2/HO-1 pathway

**Fig. 3:**
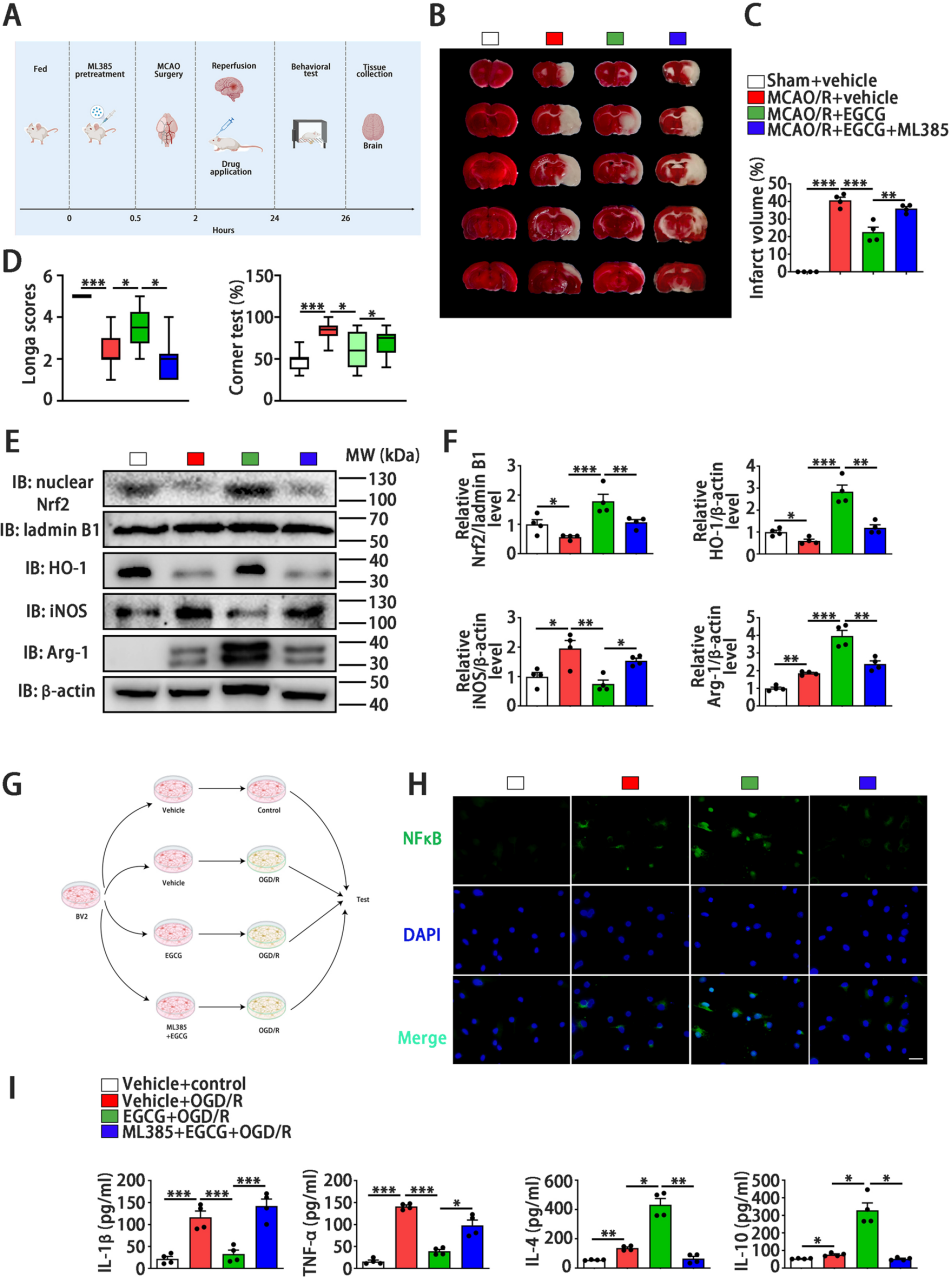
EGCG Promotes Microglial M2 Polarization via Nrf2/HO-1 Pathway. **(A)** Schematic diagram of this in vivo section. **(B**, **C)** Representative images of TTC staining and quantification of infarct volume, *n* = 4. **(D)** Modified Longa score test and Corner test, *n* = 10. **(E**, **F)** Western blot analysis of Nrf2, HO-1, iNOS and Arg-1, *n* = 4. **(G)** Schematic diagram of this in vitro section. **(H)** Immunofluorescence staining for NFκB. Scar bar = 100 μm. **(I)** The pro-inflammatory cytokines and anti-inflammatory in BV2 cells detected by ELISA assay, *n* = 4. Data are presented as mean ± SEM, and statistical analysis is performed using one-way ANOVA test followed by Tukey’s multiple comparisons test when P value of Levene test > 0.05 or Welch test followed by Dunnett T3 multiple comparisons test when the P value of Levene test < 0.05. Normality and variance are assessed via Shapiro-Wilk test and Levene’s test, respectively. ^*^ P *< 0.05*, ^**^ *P* < 0.01, ^***^ *P* < 0.001.

Nrf2, a crucial transcription factor in CIRI, also modulates microglial phenotype polarization. To uncover the link between the effect of EGCG on microglial phenotypic polarization and Nrf2-related pathways, we detected the expression of Nrf2-related proteins and pretreated BV2 cells with Nrf2-specific inhibitor ML385 (Fig. 3A). TTC staining revealed significantly larger cerebral infarct volumes in MCAO/R+EGCG+ML385 rats compared with the MCAO/R+EGCG group (Fig. 3B-C). Moreover, the ML385-treated group exhibited more severe neurological deficits than MCAO/R+EGCG group (Fig. 3D). Additionally, western blot analysis demonstrated that EGCG treatment significantly activated the Nrf2-HO-1 pathway in MCAO/R rats, concomitant with reduced expression of the M1 microglial marker iNOS and increased levels of the M2 marker Arg-1. Intriguingly, ML385 pretreatment abolished both the EGCG-mediated Nrf2-HO-1 activation and microglial phenotype switching in MCAO/R rats (Fig. 3E-F and Supplementary file 2).

We subsequently validated the role of the Nrf2/HO-1 pathway in EGCG-mediated neuroinflammatory regulation using BV2 microglial cells in vitro (Fig. 3G). We examined the translocation of phosphorylated-nuclear factor kappa B (NF-κB) p65 by immunofluorescence. The results showed that p65 was activated and translocated to the nucleus in the vehicle group; but, EGCG blocked the nuclear translocation of p65. In contrast, p65 was phosphorylated and underwent nuclear translocation in the MCAO/R+EGCG+ML385 group (Fig. 3H). Notably, ELISA revealed that ML385 pretreatment completely abrogated EGCG’s regulation on both pro-inflammatory and anti-inflammatory cytokine expression (Fig. 3I).

### EGCG ameliorate neuronal injury by mediating BV2 cells phenotype

**Fig. 4:**
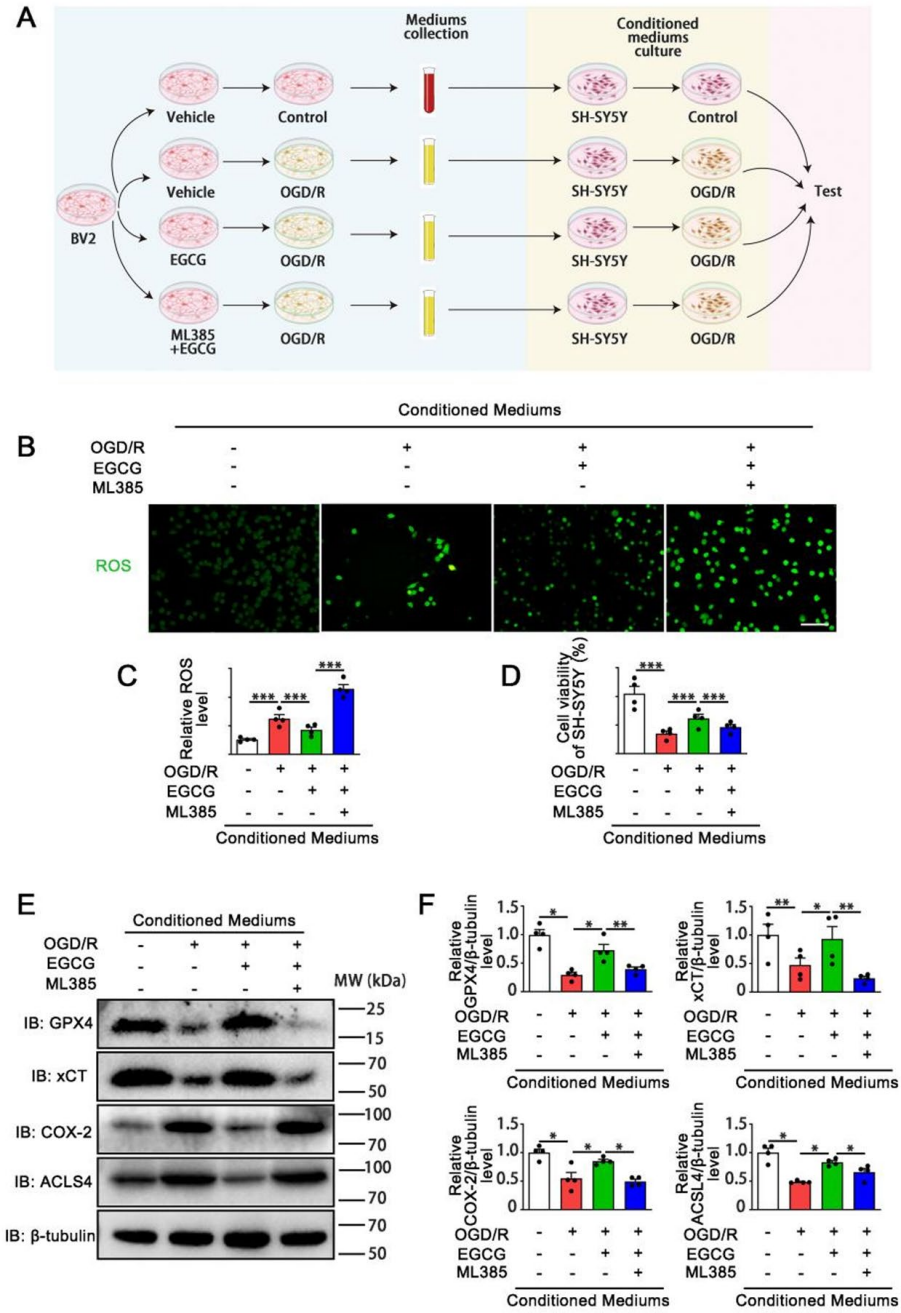
EGCG Protects SY5Y through Inhibiting Ferroptosis Induced by BV2 Cells Subjected to OGD/R. **(A)** Schematic diagram of this in vitro section. **(B**, **C)** The ROS level measured by assay kit, *n* = 4. **(D)** The cell viability of SH-SY5Y was detected by CCK-8 kit. *n* = 4/group. **(E**, **F)** Western blot analysis of GPX4, xCT, ACSL4, COX-2, *n* = 4/group. Data are presented as mean ± SEM, and statistical analysis is performed using one-way ANOVA test followed by Tukey’s multiple comparisons test when P value of Levene test > 0.05 or Welch test followed by Dunnett T3 multiple comparisons test when the P value of Levene test < 0.05. Normality and variance are assessed via Shapiro-Wilk test and Levene’s test, respectively. ^*^ P *< 0.05*, ^**^ *P* < 0.01, ^***^ *P* < 0.001.

To confirm the protective effect of EGCG against CIRI, BV2 cell supernatants from all four groups were transferred to SH-SY5Y cells (Fig. 4A). We found that the conditioned medium from the OGD/R+EGCG group significantly reduced ROS generation in SY5Y while ML385 could reverse these effects (Fig. 4B-C). Additionally, conditioned medium from OGD/R+EGCG group showed protective effect to SY5Y compared with other groups (Fig. 4D). As shown in Fig. 3E-F and Supplementary file 2, Glutathione Peroxidase 4 (GPX4) and solute carrier family 7 member 11 (SLC7A11/xCT), two critical proteins of the defense system against ferroptosis, were found decreased in the group cultured with conditioned medium from OGD/R+vehicle group. Whereas, the medium from BV2 cell pretreated with OGD/R+EGCG can rescue these two proteins in SY5Y. However, the medium from the OGD/R+EGCG+ML385 group failed to rescue GPX4 and xCT. Moreover, we found that acyl-CoA synthetase long-chain family member 4 (ACSL4) and cyclooxygenase-2 (COX-2), two important enzymes that cause lipid peroxidation in ferroptosis, were upregulated in SY5Y under the influence of the medium from the OGD/R+vehicle group. On the contrary, medium from the OGD/R+EGCG group significantly inhibited these enzymes in SY5Y. However, ML385 reversed this effect. Hence, these data verify that EGCG ameliorates the OGD/R-induced neuronal ferroptosis-like injury by promoting the microglial phenotype.

## Discussion

**Fig. 5:**
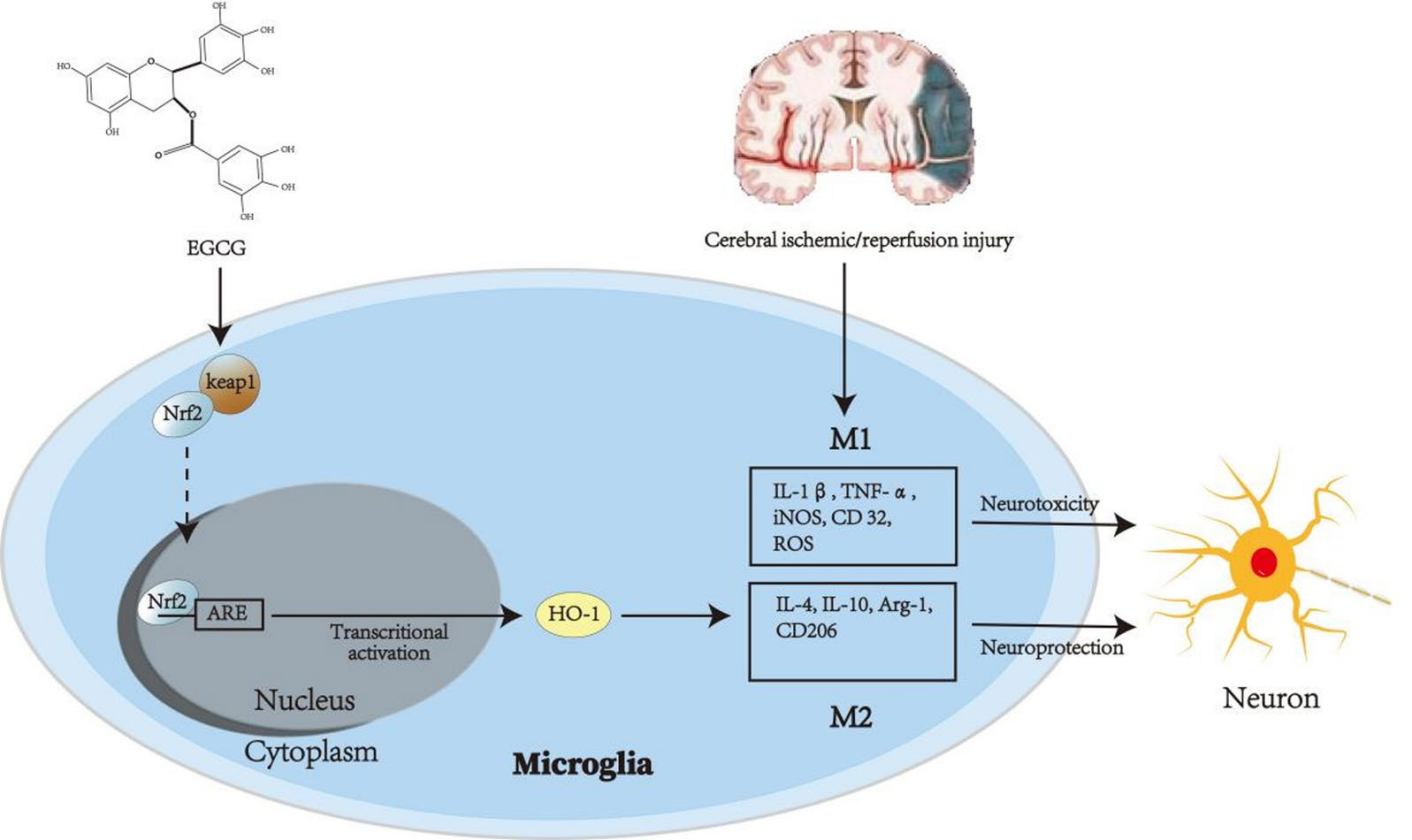
Schematic diagram of the study.

Neuroinflammation following cerebral artery recanalization remains a major therapeutic challenge in ischemic stroke. In this study, we investigated the association between therapeutic effects of EGCG and microglial phenotype switching in ischemic stroke. Our results demonstrate that EGCG exerts protective effects against CIRI, primarily through promoting microglial polarization toward the anti-inflammatory M2 phenotype. Additionally, the neuroprotection of EGCG is mediated via activation of the Nrf2/HO-1 pathway, which ultimately attenuated CIRI by suppressing NF-κB activation and ameliorating neuronal injury (Fig. 5).

EGCG is a flavonoid that tends to be catabolized during the metabolic cycle. Nanotechnology in combination with EGCG can improve its stability and aid in completely exerting effects in humans^27^. In the present study, we administered EGCG in rats by intracerebroventricular injection to ensure blood-brain barrier penetration and precise brain delivery. Interestingly, EGCG exerted protective effect on MCAO/R rats. Additionally, EGCG pretreatment decreased the M1 microglial marker CD32 level while increasing the M2 microglial marker CD206 level. Furthermore, in the present study, EGCG inhibited iNOS and upregulated Arg-1 expression both in vivo and in vitro following CIRI. Nitric oxide (NO) is a toxin that responds to ischemia and hypoxia, and is mainly induced by iNOS secreted by M1 microglia in the brain. iNOS is not present in the brain under normal physiological conditions, but is expressed following traumatic, ischemic, or inflammatory damage^28^. Arg-1, an essential enzyme that catalyzes the urea cycle, has been reported to inhibit the activation of the immune system and improve prognosis in ischemic stroke models, and is regarded as a marker of M2 microglia^29^. Moreover, ELISA analyses also confirmed that EGCG inhibited levels of M1-related inflammatory IL-1β and TNF-α, while promoted those of anti-inflammatory IL-4 and IL-10 cytokines. Hence, shifting microglia toward the M2 anti-inflammatory phenotype represents an indispensable mechanism for the protective effect of EGCG against CIRI. Moreover, EGCG also protected BV2 cells against OGD/R and increased cell viability. Therefore, the protection of microglia against CIRI plays another vital role of EGCG in the present study. Of note, there are some limitations in this study. First, BV2 cells do not fully represent all the characteristics of microglia, and future studies incorporating primary microglia are necessary. Second, as a pharmacological inhibitor, ML385 does not achieve complete silencing of Nrf2. Genetic editing techniques could provide further evidence. In summary, this study represents a preliminary exploration of the mechanism by which EGCG alleviates CIRI through the Nrf2/HO-1 pathway in microglia.

Microglia are the first resident immune cells in the CNS to respond to acute ischemic injury. However, severe ischemic stroke often leads to microglial overactivation, leading to limited neurogenesis and axon regeneration^30^. These damages are mainly caused by M1-polarized microglia that include the phagocytosis of surviving neurons and secretion of pro-inflammatory factors. In contrast, M2-polarized microglia play a role in tissue repair and neuroprotection through secretion of anti-inflammatory and neurotrophic factors^31^. In the current study, we established a severe ischemic injury in vivo model and both M1 and M2 markers drastically increased, consistent with the theory above. After ischemic stroke, injury is often accompanied by aggregation of microglia and invasion of macrophages from the peripheral blood^32^. Owing to their homology, both macrophages and microglia exhibit IBA-1. To focus on microglia, we used the mouse microglial cell line, BV2, for in vitro experiments. The BV2 cells that suffered from OGD/R were also significantly activated in vitro. Specifically, the expression of iNOS and Arg-1 was upregulated, and both pro-inflammatory (IL-1β and TNF-α) and anti-inflammatory factors (IL-4) were induced by OGD/R. These findings demonstrate that microglial phenotype modulation serves as the pivotal mechanism through which EGCG counteracts CIRI. Unlike previously reported protective effects of EGCG in ischemia, this novel mechanism provides a fresh therapeutic strategy for reperfusion therapy in ischemic stroke.

Nrf2 plays an important role in resistance to oxidative stress. Recent studies suggest that regulation of inflammation is another function of Nrf2^33^. As a major downstream protein of Nrf2, HO-1 also participates in this function^34^. After EGCG treatment, expression of Nrf2 and HO-1 was significantly upregulated, in parallel with Arg-1. NF-κB, a key factor in the classical inflammatory pathway, promotes the activation of pro-inflammatory cytokines, and activation of the Nrf2 system is critical to disrupt this cycle^35^. The present study suggested that OGD/R caused the nuclear translocation and phosphorylation of NF-κB p65. However, EGCG blocked the nuclear translocation of phospho-NF-κB p65. In contrast, EGCG failed to inhibit NF-κB p65 activation after ML385 pretreatment. ML385 also impaired the ability of EGCG to inhibit the downstream inflammatory cytokines. As ML385 significantly weakened the anti-inflammatory effect of EGCG, we further explored the influence of ML385 and Nrf2 on CIRI. These results indicate that EGCG failed to reduce the volume of cerebral infarction in MCAO/R rats after that ML385 silenced Nrf2. Besides, EGCG significantly reduced toxin levels in BV2 cells exposed to OGD/R. However, suppression of Nrf2 by ML385 significantly reduced the viability of SH-SY5Y cells. These results suggest that the neuroprotective effect of EGCG is mediated via inhibition of NF-κB to promote microglial polarization toward an anti-inflammatory phenotype, and that the Nrf2/HO-1 pathway participates in this protective effect.

Ferroptosis is a vital cell death form defined by Dixon in 2012^36^. It is characterized by intracellular polyunsaturated fatty acid (PUFA) peroxidation, resulting in the accumulation of ROS and mitochondrial failure. The regulated network of ferroptosis includes many pathways, among which amino acid metabolism, lipid metabolism, and iron ion metabolism are the critical pathways^37^. xCT and GPX4 are key proteins in the amino acid pathway and can reduce PUFA by regulating the exchange of glutamate and cystine. ACSL4 can capture PUFA (such as arachidonic acid) of cells and initiate phospholipylation. Subsequently, the phospholipid oxidase, like COX-2, will complete the oxidation of lipids, making them an element of peroxidation as well as initiating ferroptosis^38^. In the present study, the conditioned medium from the BV2 cells subjected to OGD/R significantly inhibited the neuronal expression of xCT and GPX4, which may disrupt the transport of amino acids in cells and trigger ferroptosis. The M1 macrophage related factors TNF, IL-1β, IL-6, and iNOS are associated with low GPX4 activity^39,40^. Additionally, this pro-inflammatory-ferroptosis communication may be established via prostaglandin-endoperoxide synthase 2 (PTGS2)^41^. The medium from BV2 cells subjected to OGD/R contained an amount of pro-inflammatory factors and suppressed GPX4 and xCT in SY5Y through this pro-inflammatory-ferroptosis communication. Failure of the antioxidant system leads to activation of lipid pathways, which are represented as increased expression of ACSL4 and COX-2. M2 macrophages, also known as pro-carcinogenic M2, are able to promote tumor growth and help tumor resist ferroptosis^42^. We found that EGCG significantly promoted microglial M2 polarization. The growth factors and anti-inflammatory factors involved in the medium from EGCG-treated BV2 cells rescued the SH-SY5Y and inhibited ferroptosis-like injury. On the contrary, when ML385 deactivated this phenotypic transformation, microglia-induced neuronal injury reappeared. It is generally believed that ferroptosis is a critical cell death form in CIRI^43^. However, it is rarely reported that the ferroptosis in CIRI is associated with microglia. Our study confirms this mechanism of microglia-driven neuronal ferroptosis. Of note, the investigation of neuronal ferroptosis in this study is based on results from SH-SY5Y cells treated with BV2 conditioned medium, and this model cannot fully recapitulate in vivo physiological processes. Future in vivo studies on microglia-driven neuronal ferroptosis, along with more specific assessments of ferroptosis (such as mitochondrial morphology and PUFAs), are necessary for further investigation.

Both inflammation and ferroptosis result in the production of large amounts of ROS. In fact, low level ROS is essential in cellular homeostasis^44^. However, when the ROS generation exceeds the cellular reductase system, it will cause inevasible damage. Remarkably, the medium from the EGCG-treated BV2 cells significantly inhibited the SY5Y ROS generation while ML385 reversed this effect. In summary, damages in neurons from microglia suffered OGD/R are associated with inflammation and further trigger ferroptosis-like injury, and the Nrf2/HO-1 pathway plays an important role in this mechanism. As an endpoint event, the neuroprotective effect of EGCG is ultimately achieved by inhibiting the overproduction of ROS.

Overall, this study serves as a mechanistic extension of EGCG in alleviating CIRI during the acute phase. It is worth noting that, to achieve the clinical translational potential of EGCG, several limitations in this study remain to be addressed. To maintain stable EGCG concentrations in the brain, we employed stereotaxic injection immediately after reperfusion. The translational direction of this model involves administering EGCG via the endovascular interventional system immediately after mechanical thrombectomy for acute stroke to reduce reperfusion injury risk. The translational direction of this model involves administering EGCG via the endovascular interventional system immediately after mechanical thrombectomy for acute stroke to reduce reperfusion risk. Furthermore, this study adopted a single-dose administration regimen immediately after cerebral reperfusion, as reperfusion following prolonged ischemia represents a critical time point for reperfusion-induced oxidative injury. In real-world clinical settings, the peak incidence of reperfusion injury occurs between 24 and 72 h post-surgery. Therefore, longer-term EGCG intervention or the development of sustained-release formulations holds significant importance for clinical translational prospects.

## Conclusion

The study demonstrated that microglia-related inflammation plays a crucial role in ischemic stroke and that EGCG mitigates CIRI both in vivo and in vitro by facilitating microglial polarization from the pro-inflammatory to anti-inflammatory phenotype. Furthermore, the present study revealed that the Nrf2/HO-1 axis, targeting NF-κB, is involved in the neuroprotective effects of EGCG against CIRI. These results provide valuable evidence for novel treatments for ischemic stroke during reperfusion process.

## Supplementary Information

Below is the link to the electronic supplementary material.

Supplementary Material 1

Supplementary Material 2

Supplementary Material 3

## Data Availability

Data supporting the findings of this study are available upon a reasonable request to the corresponding author.

## References

[1] Sarraj, A. et al. Endovascular thrombectomy for large ischemic stroke across ischemic injury and penumbra profiles. *Jama* **331**, 750–763 (2024). 38324414 10.1001/jama.2024.0572 PMC10851143

[2] Zhang, M. et al. Ischemia-reperfusion injury: molecular mechanisms and therapeutic targets. *Signal. Transduct. Target. Ther.* **9**, 12 (2024). 38185705 10.1038/s41392-023-01688-x PMC10772178

[3] Qin, C. et al. Signaling pathways involved in ischemic stroke: molecular mechanisms and therapeutic interventions. *Signal. Transduct. Target. Ther.* **7**, 215 (2022). 35794095 10.1038/s41392-022-01064-1 PMC9259607

[4] Faustino, J. V. et al. Microglial cells contribute to endogenous brain defenses after acute neonatal focal stroke. *J. Neurosci.* **31**, 12992–13001 (2011). 21900578 10.1523/JNEUROSCI.2102-11.2011 PMC3539822

[5] Lalancette-Hébert, M., Gowing, G., Simard, A., Weng, Y. C. & Kriz, J. Selective ablation of proliferating microglial cells exacerbates ischemic injury in the brain. *J. Neurosci.* **27**, 2596–2605 (2007). 17344397 10.1523/JNEUROSCI.5360-06.2007 PMC6672496

[6] Xiong, X. Y., Liu, L. & Yang, Q. W. Functions and mechanisms of microglia/macrophages in neuroinflammation and neurogenesis after stroke. *Prog. Neurobiol.* **142**, 23–44 (2016). 27166859 10.1016/j.pneurobio.2016.05.001

[7] Peng, X., McClements, D. J., Liu, X. & Liu, F. EGCG-based nanoparticles: Synthesis, properties, and applications. *Crit. Rev. Food Sci. Nutr.* **65**, 2177–2198 (2025). 38520117 10.1080/10408398.2024.2328184

[8] Zhao, H. et al. Efficacy of epigallocatechin-3-gallate in preventing dermatitis in patients with breast cancer receiving postoperative radiotherapy: A double-blind, placebo-controlled, phase 2 randomized clinical trial. *JAMA Dermatol.* **158**, 779–786 (2022). 35648426 10.1001/jamadermatol.2022.1736 PMC9161122

[9] Eng, Q. Y., Thanikachalam, P. V. & Ramamurthy, S. Molecular understanding of Epigallocatechin gallate (EGCG) in cardiovascular and metabolic diseases. *J. Ethnopharmacol.* **210**, 296–310 (2018). 28864169 10.1016/j.jep.2017.08.035

[10] Chattree, V. et al. A comprehensive review on modulation of SIRT1 signaling pathways in the immune system of COVID-19 patients by phytotherapeutic melatonin and epigallocatechin-3-gallate. *J. Food Biochem.* **46**, e14259 (2022). 35662052 10.1111/jfbc.14259 PMC9347991

[11] Li, S., Wang, Z., Liu, G. & Chen, M. Neurodegenerative diseases and catechins: (-)-epigallocatechin-3-gallate is a modulator of chronic neuroinflammation and oxidative stress. *Front. Nutr.* **11**, 1425839 (2024). 39149548 10.3389/fnut.2024.1425839 PMC11326534

[12] Park, D. J., Kang, J. B. & Koh, P. O. Epigallocatechin gallate improves neuronal damage in animal model of ischemic stroke and glutamate-exposed neurons via modulation of hippocalcin expression. *PLoS One* **19**, e0299042 (2024). 38427657 10.1371/journal.pone.0299042 PMC10906901

[13] Azami, S. & Forouzanfar, F. Therapeutic potentialities of green tea (Camellia sinensis) in ischemic stroke: biochemical and molecular evidence. *Metab. Brain Dis.* **39**, 347–357 (2024). 37721652 10.1007/s11011-023-01294-4

[14] Kong, X. et al. Itaconate alleviates anesthesia/surgery-induced cognitive impairment by activating a Nrf2-dependent anti-neuroinflammation and neurogenesis via gut-brain axis. *J. Neuroinflamm.* **21**, 104 (2024). 10.1186/s12974-024-03103-w PMC11034021 38649932

[15] Hao, L. et al. EGCG activates Keap1/P62/Nrf2 pathway, inhibits iron deposition and apoptosis in rats with cerebral hemorrhage. *Sci. Rep.* **14**31474. (2024). 39732956 10.1038/s41598-024-82938-y PMC11682079

[16] Cai, M. et al. Sevoflurane preconditioning protects experimental ischemic stroke by enhancing anti-inflammatory microglia/macrophages phenotype polarization through GSK-3β/Nrf2 pathway. *CNS Neurosci. Ther.* **27**, 1348–1365 (2021). 34370899 10.1111/cns.13715 PMC8504524

[17] Tao, W. et al. Magnolol attenuates depressive-like behaviors by polarizing microglia towards the M2 phenotype through the regulation of Nrf2/HO-1/NLRP3 signaling pathway. *Phytomed.: Int. J. Phytother. Phytopharmacol.* **91**, 153692 (2021). 10.1016/j.phymed.2021.153692 34411834

[18] Lei, X. et al. The novel Nrf2 activator CDDO-EA attenuates cerebral ischemic injury by promoting microglia/macrophage polarization toward M2 phenotype in mice. *CNS Neurosci. Ther.* **27**, 82–91 (2021). 33280237 10.1111/cns.13496 PMC7804925

[19] Zhang, Y. et al. Leptin alleviates endoplasmic reticulum stress induced by cerebral ischemia/reperfusion injury via the PI3K/Akt signaling pathway. *Biosci. Rep.* **42** (2022). 10.1042/BSR20221443 PMC9744719 36367210

[20] Yao, C., Zhang, J., Liu, G., Chen, F. & Lin, Y. Neuroprotection by (-)-epigallocatechin-3-gallate in a rat model of stroke is mediated through inhibition of endoplasmic reticulum stress. *Mol. Med. Rep.* **9**, 69–76 (2014). 24193141 10.3892/mmr.2013.1778

[21] Fu, Y. et al. Sublingual edaravone dexborneol for the treatment of acute ischemic stroke: The TASTE-SL randomized clinical trial. *JAMA Neurol.* **81**, 319–326 (2024). 38372981 10.1001/jamaneurol.2023.5716 PMC10877503

[22] Yuan, Y., Zhai, Y., Chen, J., Xu, X. & Wang, H. Kaempferol ameliorates oxygen-glucose deprivation/reoxygenation-induced neuronal ferroptosis by activating Nrf2/SLC7A11/GPX4 axis. *Biomolecules*10.3390/biom11070923 (2021). 34206421 10.3390/biom11070923 PMC8301948

[23] Zhong, X. et al. Epigallocatechin-3-gallate attenuates microglial inflammation and neurotoxicity by suppressing the activation of canonical and noncanonical inflammasome via TLR4/NF-κB. *Mol. Nutr. Food Res.* **63**, e1801230 (2019). 31374144 10.1002/mnfr.201801230

[24] Xie, G. et al. Asparagine endopeptidase inhibition attenuates tissue plasminogen activator-induced brain hemorrhagic transformation after ischemic stroke. *CNS Neurosci. Ther.* **31**, e70345 (2025). 40116141 10.1111/cns.70345 PMC11926568

[25] Hu, X. et al. Microglia/macrophage polarization dynamics reveal novel mechanism of injury expansion after focal cerebral ischemia. *Stroke* **43**, 3063–3070 (2012). 22933588 10.1161/STROKEAHA.112.659656

[26] Wang, Y. et al. A dual AMPK/Nrf2 activator reduces brain inflammation after stroke by enhancing microglia M2 polarization. *Antioxid. Redox Signal.* **28**, 141–163 (2018). 28747068 10.1089/ars.2017.7003

[27] Capasso, L. et al. Epigallocatechin gallate (EGCG): Pharmacological properties, biological activities and therapeutic potential. *Molecules* (Basel, Switzerland) **30** (2025). 10.3390/molecules30030654 PMC11821029 39942757

[28] Blanco, S., Muñoz-Gallardo, M. D. M., Hernández, R. & Peinado, M. The interplay between melatonin and nitric oxide: Mechanisms and implications in stroke pathophysiology. *Antioxidants (Basel, Switzerland)*10.3390/antiox14060724 (2025). 40563356 10.3390/antiox14060724 PMC12190141

[29] Liu, H. et al. Propofol improves sleep deprivation-induced sleep structural and cognitive deficits via upregulating the BMAL1 expression and suppressing microglial M1 polarization. *CNS Neurosci. Ther.* **30**, e14798 (2024). 39015099 10.1111/cns.14798 PMC11252557

[30] Xu, L. et al. Effusol ameliorates ischemic stroke by targeting NLRP3 protein to regulate NLRP3 inflammasome-mediated pyroptosis. *Phytomed.: Int. J. Phytother. Phytopharmacol.* **136**, 156253 (2025). 10.1016/j.phymed.2024.156253 39615210

[31] Du, O. et al. High mobility group box 1, a novel serotonin receptor-7 negative modulator, contributes to M2 microglial ferroptosis and neuroinflammation in post-stroke depression. *Free Radic. Biol. Med.*10.1016/j.freeradbiomed.2025.06.025 (2025). 40527447 10.1016/j.freeradbiomed.2025.06.025

[32] Franco, R. & Fernández-Suárez, D. J. P. D.J.P.i.n. Fernández-Suárez, Alternatively activated microglia and macrophages in the central nervous system. *Prog. Neurobiol.* **131**, 65–86 (2015). 26067058 10.1016/j.pneurobio.2015.05.003

[33] Choi, J. W., Im, J. H. & Balakrishnan, R. Paeoniflorin exercise-mimetic potential regulates the Nrf2/HO-1/BDNF/CREB andAPP/BACE-1/NF-κB/MAPK signaling pathways to reduce cognitive impairments andneuroinflammation in amnesic mouse model. *Biomed. Pharmacother.* **189**, 118299 (2025). 40592207 10.1016/j.biopha.2025.118299

[34] Balakrishnan, R., Kim, Y. S., Kang, S. I. & Choi, D. K. Green oat cognitaven® attenuates mild cognitive impairment by activating theCREB/BDNF/Nrf2/HO-1 pathway and modulating NF-κB/MAPK signaling. *Biomed. Pharmacother.* **189**, 118295 (2025). 40580879 10.1016/j.biopha.2025.118295

[35] Ahmed, S. M., Luo, L., Namani, A., Wang, X. J. & Tang, X. Nrf2 signaling pathway: Pivotal roles in inflammation. *Biochim. Biophys. Acta (BBA)* **1863**, 585–597 (2017). 10.1016/j.bbadis.2016.11.005 27825853

[36] Dixon, S. J. et al. Ferroptosis: an iron-dependent form of nonapoptotic cell death. *Cell* **149**, 1060–1072 (2012). 22632970 10.1016/j.cell.2012.03.042 PMC3367386

[37] Ru, Q. et al. Iron homeostasis and ferroptosis in human diseases: mechanisms and therapeutic prospects. *Signal. Transduct. Target. Ther.* **9**, 271 (2024). 39396974 10.1038/s41392-024-01969-z PMC11486532

[38] Stockwell, B. R. Ferroptosis turns 10: Emerging mechanisms, physiological functions, and therapeutic applications. *Cell* **185**, 2401–2421 (2022). 35803244 10.1016/j.cell.2022.06.003 PMC9273022

[39] Latchoumycandane, C., Marathe, G. K., Zhang, R. & McIntyre, T. M. Oxidatively truncated phospholipids are required agents of tumor necrosis factor α (TNFα)-induced apoptosis. *J. Biol. Chem.* **287**, 17693–17705 (2012). 22433871 10.1074/jbc.M111.300012 PMC3366783

[40] Katunga, L. A. et al. Obesity in a model of gpx4 haploinsufficiency uncovers a causal role for lipid-derived aldehydes in human metabolic disease and cardiomyopathy. *Mol. metabolism*. **4**, 493–506 (2015). 10.1016/j.molmet.2015.04.001 PMC4443294 26042203

[41] Friedmann Angeli, J. P., Krysko, D. V. & Conrad, M. Ferroptosis at the crossroads of cancer-acquired drug resistance and immune evasion. *Nat. Rev. Cancer* **19**, 405–414 (2019). 31101865 10.1038/s41568-019-0149-1

[42] Dai, E. et al. Autophagy-dependent ferroptosis drives tumor-associated macrophage polarization via release and uptake of oncogenic KRAS protein. *Autophagy* **16**, 2069–2083 (2020). 31920150 10.1080/15548627.2020.1714209 PMC7595620

[43] Wang, Y., Wu, S., Li, Q., Sun, H. & Wang, H. Pharmacological inhibition of ferroptosis as a therapeutic target for neurodegenerative diseases and strokes. *Adv. Sci. (Weinheim, Baden-Wurttemberg, Germany)* **10**, e2300325 (2023). 10.1002/advs.202300325 PMC10460905 37341302

[44] Goitre, L. et al. KRIT1 regulates the homeostasis of intracellular reactive oxygen species. *PLoS One* **5**, e11786 (2010). 20668652 10.1371/journal.pone.0011786 PMC2910502

