# Supplementary figures and images for: Epigallocatechin-3-gallate mitigates cerebral ischemia-reperfusion injury by promoting microglia toward M2 phenotype via Nrf2/HO-1 pathway
Source: Sci Rep. 2026 Jun 28;16:22279. doi: 10.1038/s41598-026-60227-0 (PMC13373204; doi:10.1038/s41598-026-60227-0)

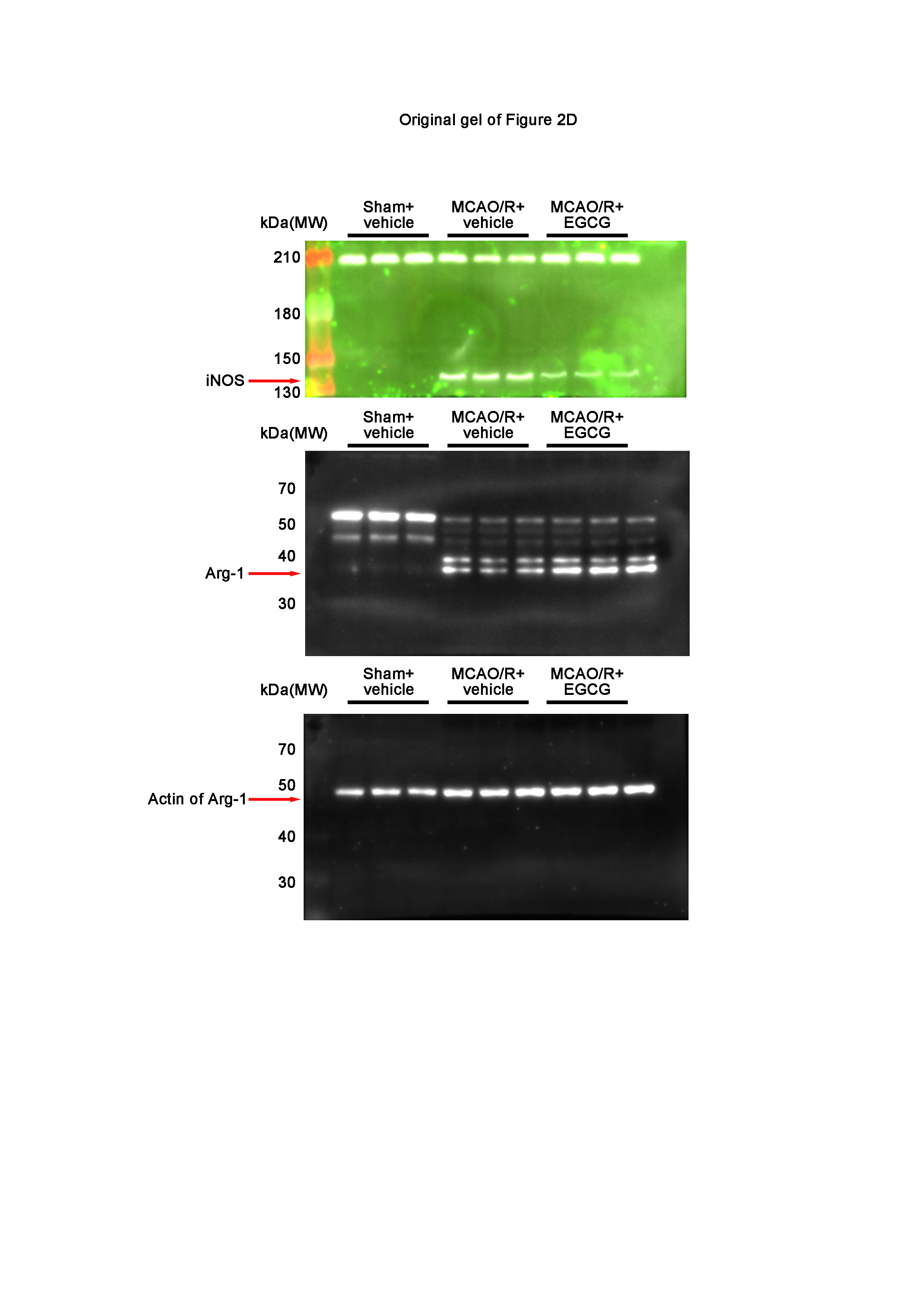

Supplement: Supplementary file 1 — Supplementary Material 1 [file 41598_2026_60227_MOESM1_ESM.jpg]

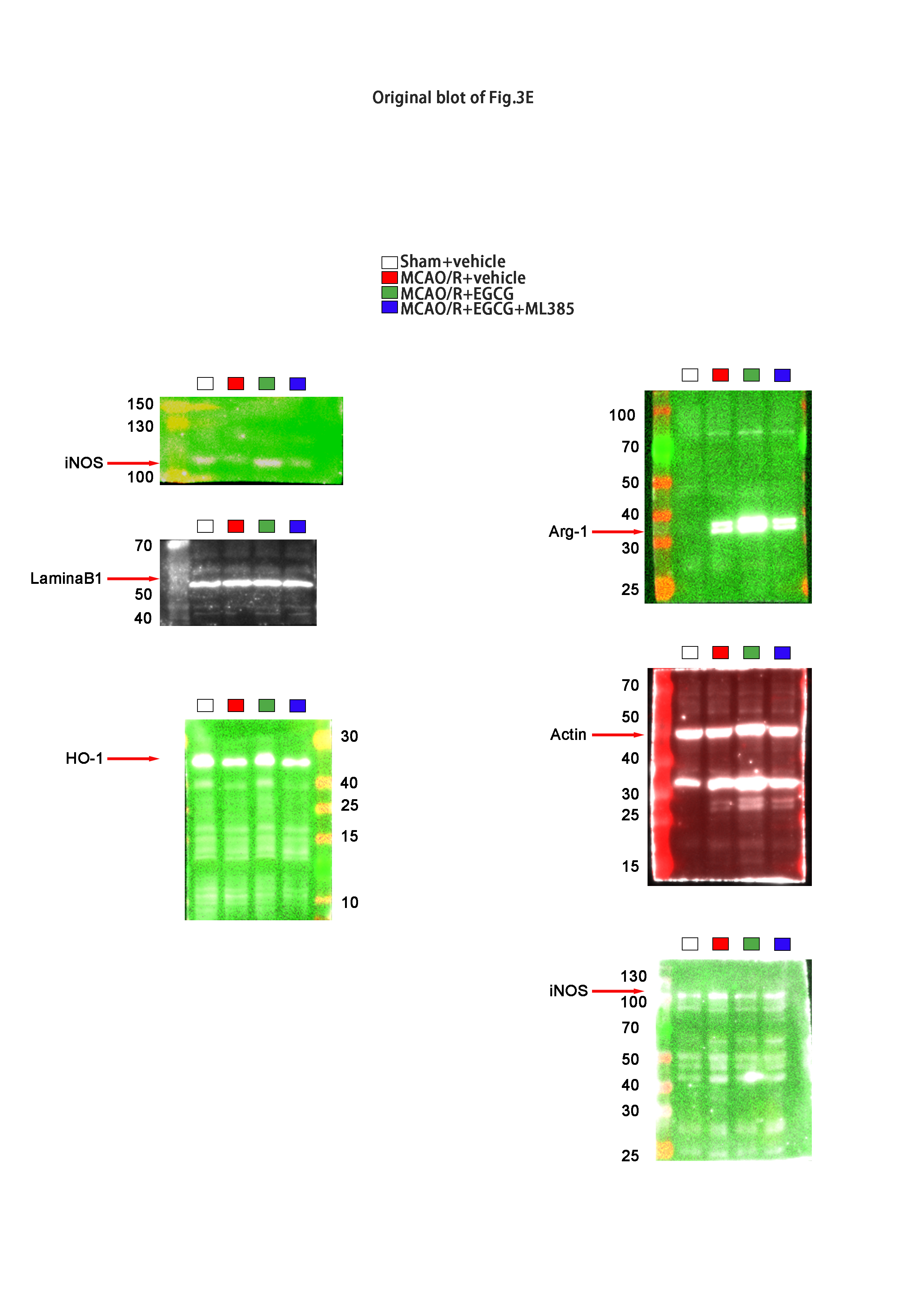

Supplement: Supplementary file 2 — Supplementary Material 2 [file 41598_2026_60227_MOESM2_ESM.jpg]

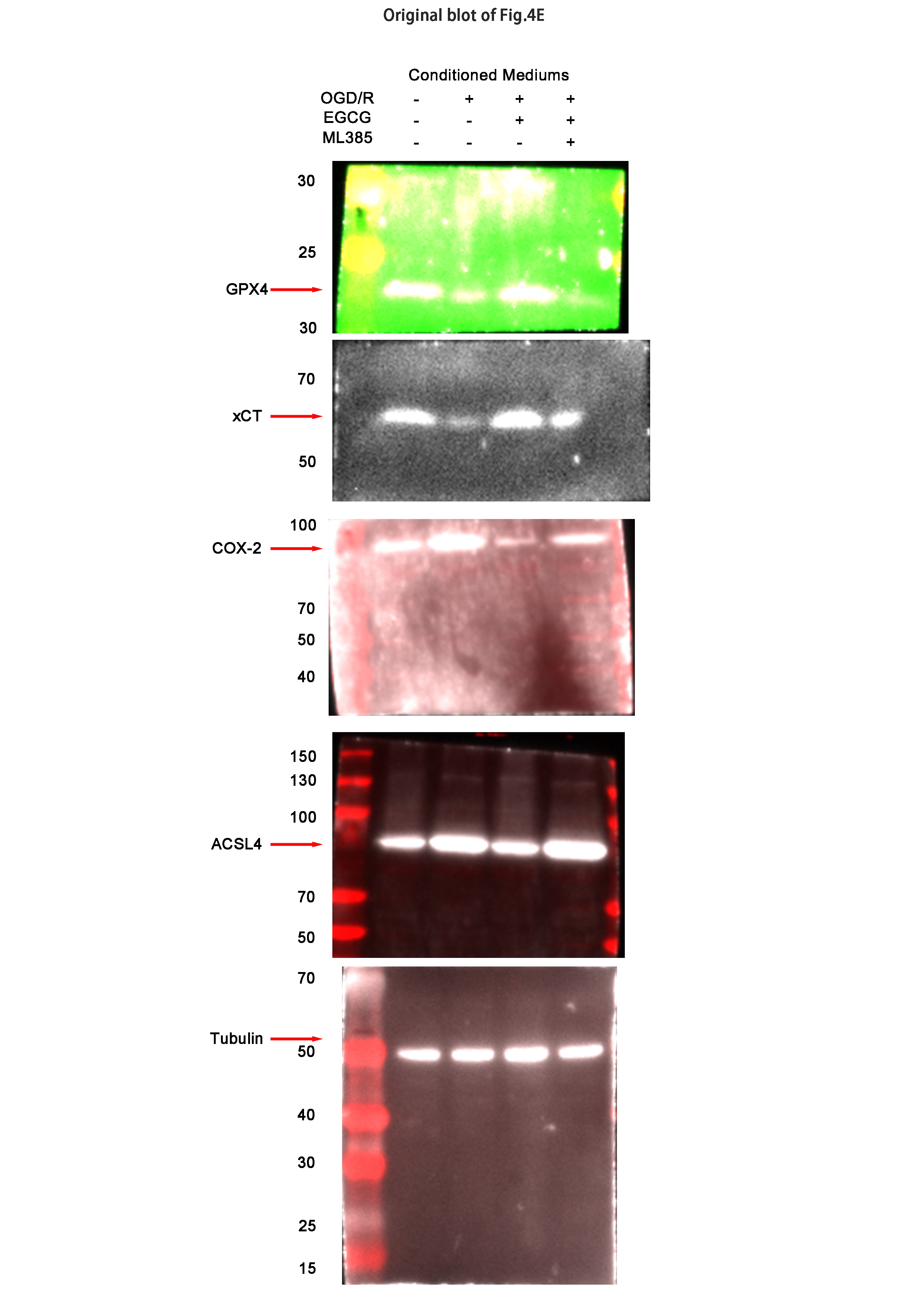

Supplement: Supplementary file 3 — Supplementary Material 3 [file 41598_2026_60227_MOESM3_ESM.jpg]
